# A Decisive Decade for Cardiovascular Health in Africa: Turning Evidence into System Design

**DOI:** 10.5334/gh.1551

**Published:** 2026-04-27

**Authors:** Johann A. C. Edjimbi, Mohamed B. Jalloh, Krutarth Kandarp Pandya, Marvellous Adeoye, Camille Lassale, Eloi Marijon, Pasquale Maffia, Bamba Gaye

**Affiliations:** 1 Alliance for Medical Research in Africa (AMedRA), Dakar, Senegal; 2 ISGlobal, Barcelona, Spain; 3 Sinai Hospital of Baltimore, Baltimore, United States; 4 Department of Hospital Medicine, Cleveland Clinic, Cleveland, Ohio, United States; 5 Institute of Public Health and Wellbeing, University of Essex, Colchester, United Kingdom; 6 Hospital del Mar Medical Research Institute (IMIM), Barcelona, Spain; 7 Consortium for Biomedical Research—Pathophysiology of Obesity and Nutrition (CIBEROBN), Instituto de Salud Carlos III, Madrid, Spain; 8 Universitat Pompeu Fabra (UPF), Barcelona, Spain; 9 INSERM U970, Paris Cardiovascular Research Centre (PARCC), 75015 Paris, France; 10 Division of Cardiology, Georges Pompidou European Hospital, 75015 Paris, France; 11 School of Infection & Immunity, College of Medical, Veterinary and Life Sciences, University of Glasgow, Glasgow, United Kingdom; 12 Department of Pharmacy, School of Medicine and Surgery, University of Naples Federico II, Naples, Italy; 13 Africa-Europe CoRE in Non-Communicable Diseases & Multimorbidity, African Research Universities Alliance (ARUA) & The Guild of European Research-intensive Universities, Glasgow, United Kingdom; 14 Department of Physiology, Cheikh Anta Diop University, Dakar, Senegal; 15 Department of Biomedical Informatics, Emory University, Atlanta, Georgia, United States

**Keywords:** Cardiovascular disease, Africa, Health systems, Universal health coverage, Health financing, Chronic disease management

## Abstract

Cardiovascular disease is now a leading cause of premature mortality across Africa and is accelerating faster than the capacity to prevent, detect, and manage chronic illness. Most patients still engage with the health system only when heart failure, stroke, or ischemic disease is advanced, reflecting a legacy architecture designed primarily to confront acute infections. At the same time, multiple African countries have demonstrated that high-impact cardiovascular care can be delivered at scale when services are organized around primary and district facilities, supported by clear protocols, continuous supply of essential medicines, workforce development, and access to remote specialist expertise. Global experience, including major reforms in Brazil and Thailand, shows that population-level gains arise from deliberate health system design. Africa now stands at a turning point. By embedding cardiovascular disease prevention and treatment within national strategies for universal health coverage and by aligning financing and service delivery with the realities of chronic care, the region can prevent millions of avoidable deaths. The opportunity to define a different future for cardiovascular health is within reach and must be acted upon with urgency and coherence.

## Central Illustration

**Figure:**
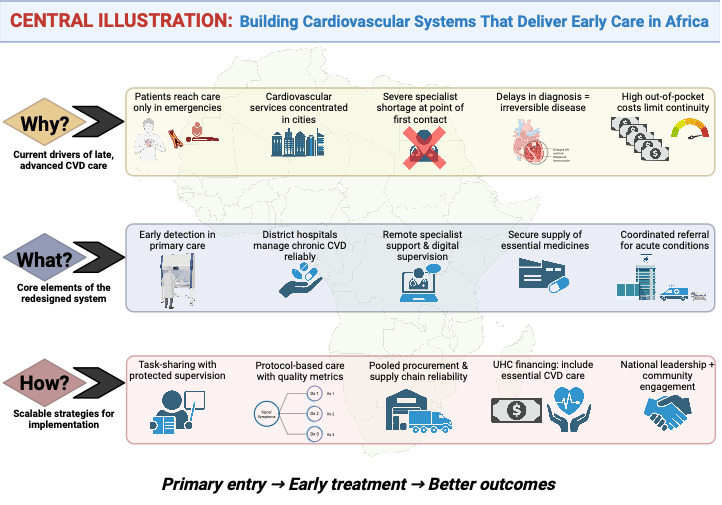


## Introduction

Cardiovascular disease (CVD) has become the leading driver of mortality across Africa, reshaping the clinical landscape of a continent long defined in global health discourse by infectious threats (1). The consequences are visible in emergency units overwhelmed by late-stage heart failure and stroke, and in primary care systems struggling to respond to a rise in chronic cardiometabolic illness that they were never structured to manage. Preventable CVD deaths are occurring earlier in life than in any other world region, translating into a profound loss of productive years and widening disparities in healthy aging (1).

This is a transition taking place at speed. Rapid urbanization, dietary shifts, and population aging are accelerating cardiometabolic risk more quickly than the capacity to detect and manage the disease is expanding (2). At the same time, persistent limitations in surveillance and vital registration obscure the full extent of the burden, leaving cardiovascular priorities underrepresented in national planning and international investment decisions (3). In many countries, patients first encounter the formal health system only when the disease is advanced, a pattern that reflects system design rather than patient behavior (3,4).

Despite this trajectory, the determinants of Africa’s CVD burden are not immutable. Across the continent, clinicians and health ministries have developed pragmatic strategies that demonstrate how high-impact cardiovascular care can be delivered in primary settings with targeted specialization, strong supervisory networks, and smart deployment of digital tools (4,5,6). These examples are not exceptions to the rule; they are signals of a direction that deserves scale.

The coming decade represents a pivotal moment: whether cardiovascular disease becomes the dominant and destabilizing health challenge in Africa, or whether the innovations already emerging across its health systems are leveraged to change the region’s long-term trajectory.

## A System Built for Yesterday’s Threats

Africa’s health systems achieved remarkable gains by organizing around the acute infectious diseases that once dominated mortality. Integrated management of childhood illness and expanded HIV services saved millions of lives and strengthened primary care platforms. But the same architecture, built for short-episode conditions and crisis response, is now struggling to absorb the rising burden of chronic cardiovascular disease (3,7). The result is a landscape in which prevention and early management are inconsistently delivered, and cardiovascular care often begins only after irreversible damage has occurred (1).

This misalignment is compounded by service concentration in urban referral hospitals. Outside major cities, access to timely diagnostics such as electrocardiography, basic echocardiography, and biomarker testing remains limited, leading to avoidable delays in recognizing acute coronary syndromes and decompensated heart failure (3,6). Rapid transfers through tiered systems of care are uncommon; instead, patients often circulate across loosely connected facilities, losing critical time as disease progresses (3).

Workforce distribution reinforces these delays. The severe shortage of cardiovascular specialists and the uneven deployment of diagnostic infrastructure place the responsibility for recognizing and managing CVD squarely on primary facilities that are neither staffed nor equipped for it (4,8). Remote supervisory models have begun to demonstrate that real-time support from experienced clinicians can expand the reach of high-quality diagnostic decision-making beyond tertiary centers, but these efforts remain limited in scale (4,6,8).

Together, acute care design, urban dependency, and weak referral continuity explain why the rising cardiovascular burden in Africa manifests so powerfully as late-detected, late-treated disease. As demographic and epidemiological pressures intensify, aligning system capabilities with the realities of cardiovascular care is no longer optional; it is foundational to improving survival and compressing morbidity.

## Signals of Progress

Notwithstanding the challenges, several African health systems have demonstrated that cardiovascular care can be reorganized to achieve earlier diagnosis and more durable outcomes. In Ghana, community-embedded hypertension programs have shown that treatment intensification and longitudinal follow-up can be delivered effectively through nurse-led care within primary health systems, with meaningful improvements in blood pressure control among adults who remain in care (9). In Rwanda, district hospitals have assumed responsibility for longitudinal management of heart failure and rheumatic heart disease, reducing dependence on tertiary centers and allowing patients to access specialty care without the delays and financial burden associated with travel to the capital (10,11).

Similar developments are emerging elsewhere. In Uganda, remote cardiovascular consultation and tele-diagnostic support have enabled clinicians in rural facilities to evaluate suspected heart disease in real time, rather than waiting for specialist outreach (6). In Cameroon, telemedicine-supported hypertension care has led to earlier treatment adjustments and clinically significant improvements in blood pressure control, demonstrating that targeted digital connectivity can partially compensate for limited specialist availability in rural districts (12).

Although each of these efforts reflects a different operational approach, they converge on a shared principle: cardiovascular care improves when primary-level facilities are empowered to detect and manage chronic disease with reliable supervisory and referral pathways. These initiatives show that technology, when coupled with clinical governance and defined care protocols, can move diagnosis closer to the point of first contact. They also show that continuity is achievable when care is organized around patient journeys rather than the episodic logic of acute care models.

These advances remain modest in geographic reach compared with the scale of cardiovascular need across the continent, yet their significance lies in the models they introduce, models that make cardiovascular care feasible where it was previously unattainable. They provide evidence that Africa’s trajectory is not predetermined by resource limitations, but can be reshaped through system design that treats cardiovascular disease as a core function of primary health care rather than a specialized exception.

## Designing for Scale

The progress now visible across parts of Africa demonstrates what can be achieved when cardiovascular care is brought closer to where people live, yet the impact of these models will remain local unless the health system itself is reoriented to ensure that early detection and chronic management are delivered reliably, not exceptionally. This requires making cardiovascular care a core function of the health system rather than a specialist service dependent on geography, individual champions, or temporary support (3).

Several regions outside Africa offer relevant lessons for how scale can be achieved through structural alignment. In Brazil, the national Family Health Strategy (FHS) has been associated with reductions in cardiovascular and cerebrovascular mortality and decreased hospitalizations for ambulatory-care-sensitive conditions, reflecting the impact of a community-based primary care model with broad coverage and continuity of care (13). A pilot within FHS demonstrated that embedding hypertension management, including follow-up and community health-worker involvement through primary care units, contributed to improved risk factor control (14). Other Brazilian city-level initiatives under the CARDIO4Cities initiative also show how urban public health reforms, when linked to evidence-based protocols and system redesign, can significantly improve hypertension detection, treatment, and control at scale (15).

Applying this principle in Africa means defining what cardiovascular care should look like at each system level. Primary facilities must consistently identify risk, begin treatment, and ensure follow-up. District-level hospitals must serve as the loci of advanced chronic care, with the capability to diagnose and stabilize heart failure, arrhythmias, and acute coronary syndromes. Tertiary centers should support this network through consultation, complex intervention, and clinical governance, rather than serving as the de facto point of entry (4).

Redistributing expertise in this way requires sustained investment in workforce development, supervision, and reliable supply chains, so that technical capacity becomes durable rather than episodic. The potential of such redistribution is supported by global frameworks emphasizing that payment and financing mechanisms significantly shape health equity outcomes; reforms to financing systems ensuring essential cardiovascular services are included in publicly funded benefit packages can increase access and continuity for disadvantaged populations (16).

Financing mechanisms determine whether such a system can endure. Where cardiovascular services depend on out-of-pocket spending, patients tend to present only when the disease is advanced. By contrast, settings that integrate essential cardiovascular interventions into public financing and benefits packages have shown greater resilience of service continuity and improved outcomes at the population level (3).

For Africa, the task ahead is to convert promising approaches into a standard of practice. The evidence is no longer theoretical: earlier diagnosis and better outcomes are attainable when care is structured to support them. The challenge is to ensure that these gains do not remain confined to specific districts or programs, but instead define how cardiovascular care is delivered across the continent.

The feasibility of scaling these models is ultimately determined by how health systems are financed. In sub-Saharan Africa, total health spending per capita remains the lowest globally (US$92 in 2021), while government health expenditure represents only about 7% of total government spending, compared with over 12% in other regions (17). Additionally, external assistance accounts for more than one-third of total health expenditure, and out-of-pocket payments frequently exceed 30%, leaving a narrow fiscal base to support longitudinal chronic care (17).

Beyond the level of spending, the structure of financing further constrains scale. Separate funding streams for formal sector insurance, community-based schemes, donor-supported programs, and ministry budgets often coexist within the same system without financial integration. This fragmentation limits the redistribution of prepaid resources and reduces purchasing efficiency. Across the World Health Organization (WHO) African Region, 33 of 43 countries report ongoing or planned reforms specifically targeting revenue raising, pooling, purchasing, and public financial management (18).

These realities imply that cardiovascular scale-up depends less on expanding services than on how existing and new resources are organized. The first priority is to protect prepaid public funds for essential services. This means ensuring that limited government and donor resources are explicitly allocated to guarantee the availability of blood pressure measurement, first-line antihypertensive and heart-failure medicines, and basic diagnostics at primary and district levels before additional services are considered. Without this protection, households remain exposed to out-of-pocket spending for the very services that prevent advanced disease (17).

A second priority is to create predictable domestic revenue streams capable of supporting chronic care over time. Several countries in the region are already expanding excise taxes on tobacco and alcohol and exploring solidarity levies as part of broader health financing reforms (18,19). These mechanisms do not generate large budgets overnight, but they provide stable, recurrent funding that can sustain longitudinal care rather than episodic emergency responses.

A third requirement is to consolidate fragmented financing pools into larger risk pools. When funding remains divided across formal sector insurance schemes, community-based arrangements, donor programs, and ministry budgets, prepaid resources cannot be redistributed efficiently across populations. Integrating these streams allows healthier and wealthier groups to subsidize higher-need populations, which is essential for financing chronic disease management at scale (18).

Finally, financing must be linked to purchasing mechanisms that support continuity of care alongside acute care. Acute cardiovascular events will always require hospital-based management, but when financing does not support early detection and longitudinal treatment at primary and district levels, hospitals become the default point of entry for advanced disease. Redirecting pooled funds toward services that provide ongoing hypertension and heart-failure management allows health systems to reduce avoidable acute presentations while preserving the essential role of hospital care (19).

## A Decisive Decade

A decisive response to cardiovascular disease must also include population-level prevention and system intelligence. Tobacco control, salt reduction, and promotion of healthy diets remain among the most cost-effective interventions available, yet prevention and public health receive a small fraction of total health spending (19). Development of context-specific cardiovascular guidelines and investment in registries and digital health systems are essential to measure burden, audit care delivery, and connect financing reforms to outcomes.

Africa’s health systems are entering a period in which the trajectory of cardiovascular disease will be shaped by decisions made now. The growing burden of heart failure, stroke, ischemic heart disease, and advanced hypertension will either force systems into perpetual crisis response or compel a reorganization that anticipates chronic need (1,2,3). The examples emerging across the continent show that meaningful improvement is possible when cardiovascular care is delivered where patients live, supported by reliable pathways for escalation and continuity (4,5,6,9,11,12).

The coming decade offers a narrowing window in which to prevent a widening gap in life expectancy and healthy aging between Africa and the rest of the world (1,3). Achieving this will not require mirroring the architectures of high-income countries, but rather scaling models that are already demonstrating effectiveness in African contexts: distributed diagnostic capability, task-sharing under supervision, secure access to essential medicines, and referral coordination that treats time as a determinant of survival. These are the building blocks of a cardiovascular system that is both realistic and transformative.

The global success stories of the past thirty years in reductions in cardiovascular mortality driven by prevention, timely care, and organized delivery systems were the result of policy decisions that treated cardiovascular disease as an urgent priority. Africa now stands at a similar threshold. If cardiovascular health is placed at the center of system strategy, the continent can avert millions of avoidable deaths and establish a future in which cardiovascular care is not a privilege but a routine part of health care.

The direction is clear. The pace must now match it.
